# New concept of pump-less forward osmosis (FO) and low-pressure membrane (LPM) process

**DOI:** 10.1038/s41598-017-15274-z

**Published:** 2017-11-06

**Authors:** Sung-Ju Im, Jungwon Choi, Sanghyun Jeong, Am Jang

**Affiliations:** Graduate School of Water Resources, Sungkyunkwan University (SKKU), 2066, Seobu-ro, Jangan-gu, Suwon-si, Gyeonggi-do, 16419 Republic of Korea

## Abstract

We tested the possibility of energy-saving water treatment methods by using a pump-less forward osmosis (FO) and low-pressure membrane (LPM) hybrid process (FO-LPM). In this pump-less FO-LPM, permeate migrates from the feed solution (FS) to the draw solution (DS) through the FO membrane by use of osmotic pressure differences. At the same time, within the closed DS tank, inner pressure increases as the DS volume increases. By using the DS tank’s internal pressure, the LPM process works to re-concentrate the diluted DS, maintaining the DS concentration and producing clean water. In this study, a polymer - polystyrene sulfonate (PSS) was used as a draw solute. Based on the results of each individual portion of the process, the optimal range of the PSS DS was determined. The performance of the pump-less FO-LPM process was lower than that of a single process; however, we observed that the hybrid process can be operated without a pump for regeneration of a diluted DS. This research highlights the feasibility and applicability of pump-less FO-LPM processes using a polymeric DS for water treatment. Additionally, it is suggested that this novel process offers a breakthrough in FO technology that is often limited by operation and management cost.

## Introduction

Demand for sustainable supplies of high-quality drinking water is increasing due to increased industrialization, urbanization, population growth and global warming^1,2^. Consequently, the need for improved water treatment processes is also increasing. Among the various existing water treatment processes, membrane separation processes are one of the most applicable technologies. However, membrane processes are prone to the following limitations: membrane fouling, the need for reliable cleaning and pre-treatment methods, and relatively high operation and maintenance (O&M) costs^3,4^. To address these issues, many studies have focused on hybrid processes that use modeling, fabrication, case studies, and system development^5–7^.

**Figure 1:**
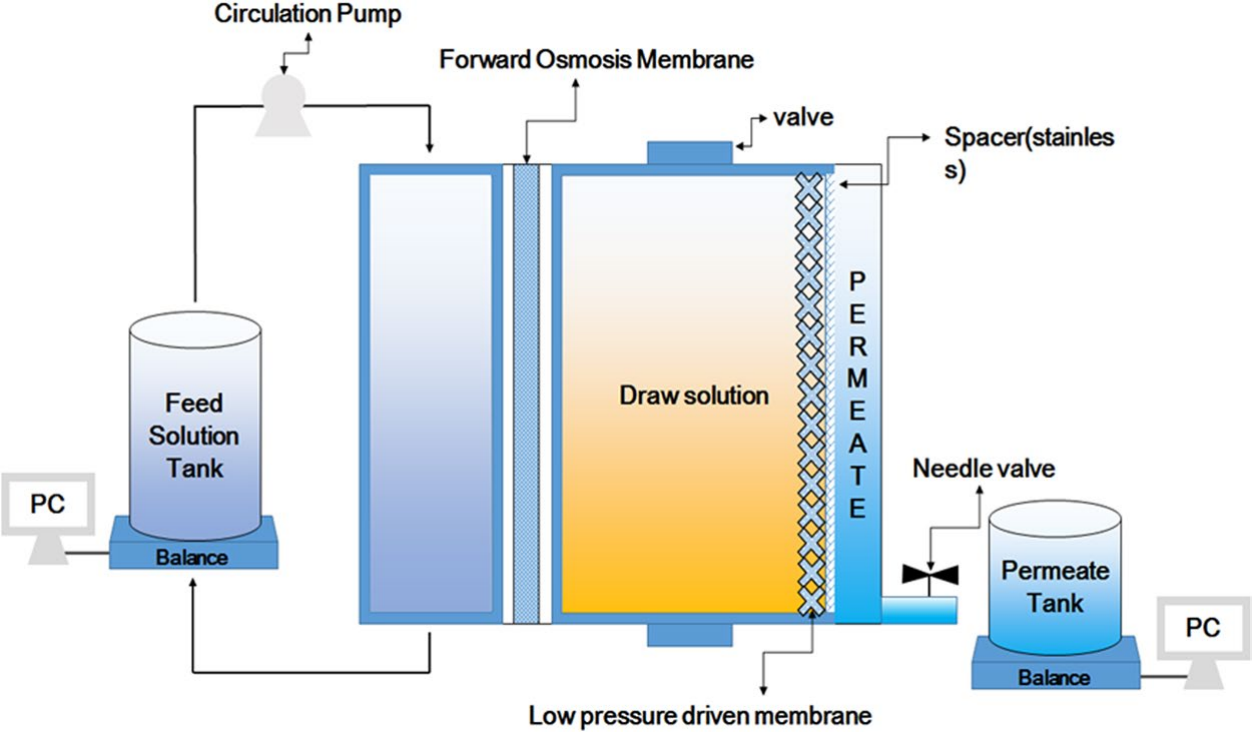
A conceptual diagram of FO-LPM system.

A hybrid system is defined as a combination of two or more single processes. Some of the advantages offered by hybrid systems include lower energy consumption rates, higher rejection rates, lower fouling propensities and enhanced process efficiency (low operating costs, etc.) compared to single processes^3,8,9^. Still, operational costs, including the cost of energy and the maintenance issues inherent in membrane processes are some of the main limitations for stand in the water treatment industrial area. The pump itself is also a significant factor in terms of costs, both in terms of operation and maintenance costs as well as overall energy costs for membrane separation processes^2,3,10^. In order to address these issues, a pump-less osmotic-driven membrane and low pressure-driven membrane (FO-LPM) hybrid process was designed for the first time. The FO-LPM system concept is presented in Fig. 1.

The FO-LPM system consists of three parts. The first part is the feed solution (FS), which is the target of the treatment system. The second part consists of the draw solution (DS) within a closed system and is what is used as the feed water (FW) for the LPM system. The tank is operated in a closed system with permeation from the FS to the DS serving to increase the volume of the DS. Thirdly, the LPM regenerates the diluted DS and produces the final product: water. As mentioned earlier, the main purpose of the FO-LPM hybrid process is to make use of the pressure that is generated by the increased volume of DS. This pressure becomes the necessary energy used to operate the LPM process; thus precluding the need for a pump to regenerate DS. Therefore, we called volume retarded osmosis (VRO) in this manuscript. However, PRO process uses potential energy to be converted to electrical energy by electric generator. In addition, high pressure pump is required to reach the minimum pressure for PRO operation. Therefore, FO-LPM system is completely different concept from PRO process. Moreover, in comparison to other hybrid systems, such as FO-reverse osmosis (RO) hybrid systems and membrane distillation (MD)-nanofiltration (NF) hybrid systems, the new FO-LPM hybrid system can reduce energy consumption with the operation of the LPM process since a regeneration pump is not required. Generally, LPM processes such as tight microfiltration (MF), ultrafiltration (UF), and loose NF are operated using low pressure, ranging from 1 to 10 bar^11–13^. Generally, inorganic salts based DSs have high osmotic pressures and water flux and are easily removed by RO.

The FO-LPM hybrid system enables the regeneration of the DS, however, as shown in previous studies, the regeneration is only possible if polymers are used as the DS. This is because the LPM cannot remove monovalent or polyvalent ions, which are much smaller than the polymers. Furthermore, use of polymers as the draw solute offers many advantages in regenerating DS for the following reasons: (1) low energy costs for DS regeneration, (2) low reverse salt flux, and (3) relatively high water flux. EDTA sodium salts, hexavalent phosphazene salts and polyaspartic acid sodium salt were successfully regenerated in previous studies using LPM with pressures under 10 bar^12–16^. An ideal DS should have following characteristics: (1) high osmotic pressure, (2) minimal reverse draw solute, (3) easy regeneration, (4) nontoxicity, (5) high water solubility, (6) low viscosity, and (7) low costs. From previous studies, PSS was found to satisfy these conditions. PSS can generate high water flux and low reverse salt diffusion and it can be recovered using the LPM process at 2 bar. Previous studies also noted that producing potable water through FO requires as much energy as the conventional RO process, since the energy required to overcome osmotic pressure does not change. This means that there is no future for FO if ionic salts are used as the DS. However, as mentioned above, polymers are larger and thus can be removed by LPM. As one of the polymers, PSS showed potential for use as a DS, and offers some distinct advantages suggesting PSS is a better DS than other polymers. These advantages include its non-toxicity and its negative charge. Currently, PSS is used as a potassium binder in acute and chronic kidney disease for people with hyperkalemia. Additionally, due to its negative charge, it is repelled from the membrane, which minimizes the RSF^13^.

In this study, the pump-less FO-LPM hybrid system was developed with the use of a large size polymer (polystyrene sulfonate, PSS) to test the feasibility and potential as a DS.

## Results

### Single performance of FO and LPM according to operation parameters

**Figure 2:**
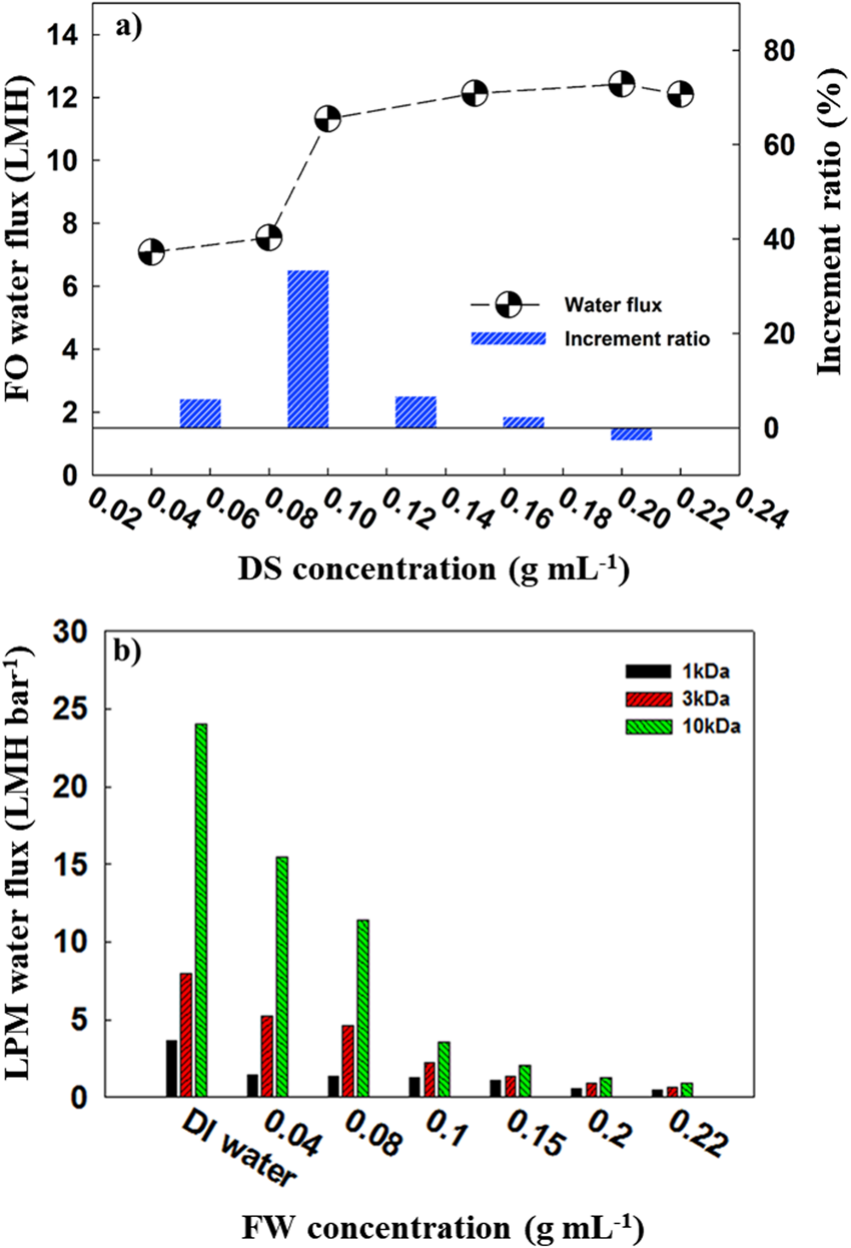
(**a**) FO water flux relative to the DS concentration, and (**b**) LPM water flux relative to the FW concentration and value of MWCO.

In a FO process, the properties and concentrations of a DS have a direct influence on the solution’s osmotic pressure and subsequently the performance of the FO process. Draw solute characteristics, such as solubility, viscosity, mobility, and its diffusion coefficient also affect the FO performance^17^. Here, we tested six different concentrations of PSS (0.04, 0.08, 0.10, 0.15, 0.20 and 0.22 g mL^−1^) to evaluate the water flux in the FO process using deionized (DI) water as the FS. The water flux values for the FO process were plotted as a function of DS concentrations of PSS (Fig. 2a). The water flux at 0.04 g mL^−1^ is about 7 Lm^−2^h^−1^ (LMH) and increases as the concentration of PSS increases. However, the water flux of the FO process was not found to have a linear relationship with PSS concentration, meaning that the water flux does not increase in line with PSS concentration increases. Additionally, the flux reaches its maximum at 0.20 g mL^−1^ and then starts to decrease. This non-linearity results from high viscosities and low diffusion coefficients, which are consequences of high concentration for PSS. The increment ratio, which is an increment between each concentration, peaks between 0.08 and 0.10 g mL^−1^, and becomes negative between 0.20 and 0.22 g mL^−1^. A highly concentrated DS results in high viscosity and a low diffusion coefficient, thus a low increasing rate of FO water flux was expected as a result of increased DS concentration. Previous studies on polymer-based draw solute reported that a high viscosity of the DS not only led to high energy consumption for fluid transport through the membrane but also resulted in a severe internal concentration polarization (ICP)^12,13,18–21^. This ICP effect takes place in the porous support layer of the asymmetric FO membrane^6,10,17,18^. Figure 2b shows the water fluxes of the UF membranes with three different molecular weight cut-offs (MWCOs) (1, 3 and 10 kDa) for filtration of the PSS solution at different concentrations. The water flux of LPM decreased as PSS concentrations increased. Water flux decreased from 15.50 to 0.94, from 5.24 to 0.63 and from 1.14 to 0.50 LMH bar^−1^ for 10, 3 and 1 kDa UF membranes, respectively. These results indicate that the concentration of PSS solution and the membrane’s MWCO are critical factors for LPM performance. This may be due to the CP layer and deposits of PSS on the LPM surface at high pressure affecting the performance of the LPM process. Additionally, high concentrations of PSS can easily permeate through the LPM, leading to a reduced rejection rate due to diffusion. The transport of PSS through the LPM that is affected by convection and/or diffusion according to the operating conditions, is represented by J_0_/k ratio. The J_0_/k ratio is represented by the water flux. The J_0_ and k represent initial permeate flux of membrane process and mass transfer coefficient, respectively. In addition, J_0_/k represents the solution rejection rate. There have been many previous researches related to membrane process used J_0_/k ratio to determine MWCO and rejection values^18,19^. Therefore, concentration differences between the FW of LPM and permeate promotes PSS molecules passing through the LPM^20^.

PSS solutions also contain molecules with different molecular weights (MW) (average MW ~70,000 g mol^−1^) and sizes. For PSS molecules with lower MWs and smaller sizes, it is relatively easy to penetrate through the pores of the LPM membrane compared to PSS molecules with higher MWs at larger sizes. Additionally, the use of highly concentrated PSS solutions in the operation of the LPM process requires higher pressures to get similar permeate flux. In the case of dead-end LPM cells, the fluid is pressed against the membrane, creating greater pressure that compresses the fouling layer, which could, in turn, lead to a more severe flux decline.

### Pump-less FO-LPM performance

**Figure 3:**
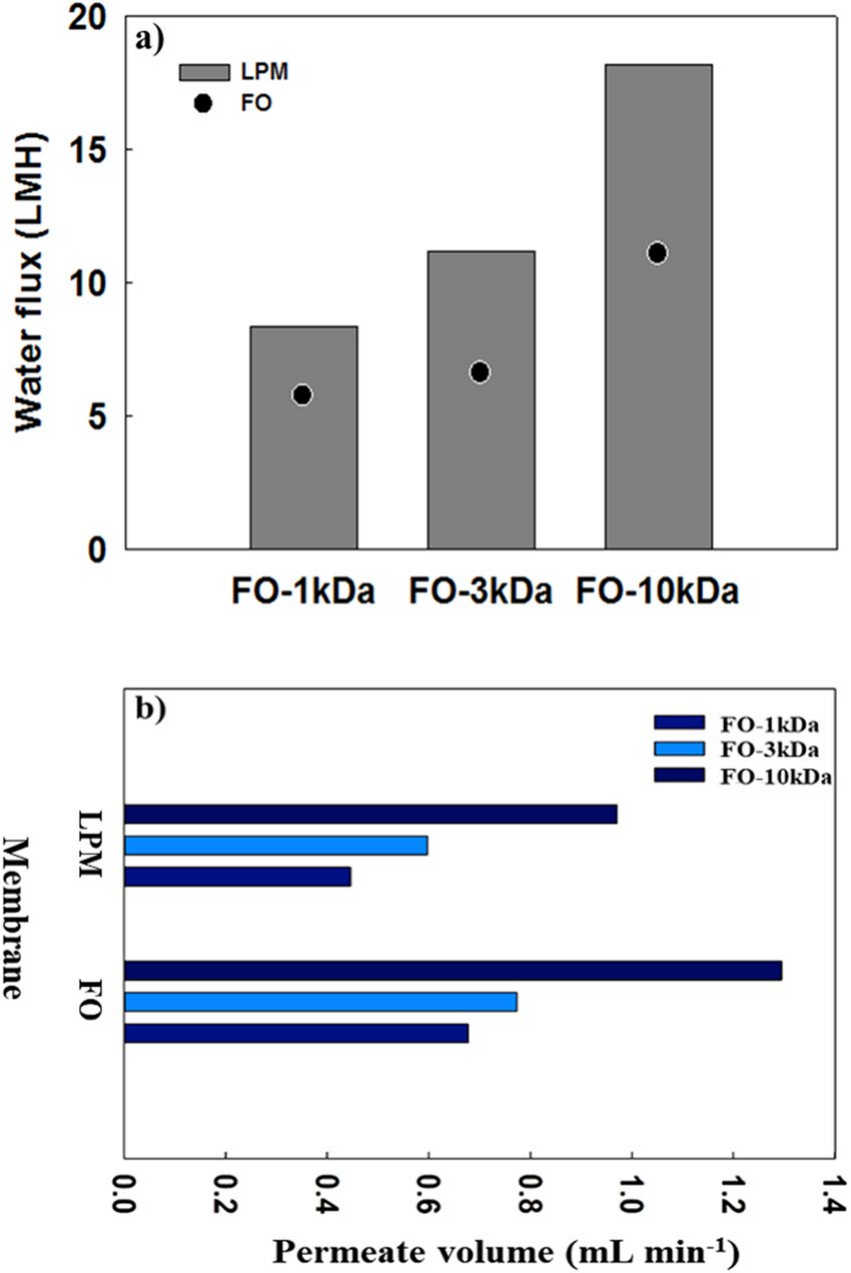
(**a**) Water flux of FO-LPM system, and (**b**) Permeate volume of FO-LPM system.

Based on the results from the individual FO and LPM processes, it was expected that the FO-LPM process would the best performance for PSS concentrations ranging between 0.10 and 0.15 g mL^−1^. Figure 3a shows water fluxes for both FO and LPM process with PSS concentration at 0.15 g mL^−1^. In this system, the DS was almost completely rejected by the LPM membrane used in the experiment as a result of the PSS average MW of 70,000 g mol^−1^. Previous studies have reported that rejection rates of PSS were from 99% to 89% at concentrations of PSS from 0.01 to 0.07 g mL^−1^ using 5 KDa UF membrane^13,21^. In this experiment, the rejection was also shown to be over 90% when 1 kDa UF membrane was used. The initial FO water flux was higher when a 10 KDa UF membrane was used in the LPM part of the process: FO water flux was 11.1 LMH, which was higher than when 3 and 1 KDa UF membranes were used in the LPM portion of the process (6.63 and 5.80 LMH, respectively). When the loose membrane (10 KDa) was used in the LPM portion, water flux for both FO and LPM processes was high. This may be because the low MWCO LPM can be operated at a relatively low operating pressure. It is also shown that increasing DS volume, resulting from the addition of permeate to the LPM portion through the FO process, subsequently increased the pressure allowing the operation of FO-LPM since the LPM process can be operated with relatively low pressure. In the case of FO-LPM process, driving force of LPM part was inner pressure of closed DS tank resulting from the increased DS volume which was generated by the permeation from the FS to the DS. However, inner pressure of DS tank has an adverse effect on the performance of FO process because the inner pressure affects both FO and LPM sides. When 10 kDa LPM membrane is used for LPM part of FO-LPM process, LPM part can be operated at a lower pressure compared to relatively tight membranes. In other word, LPM can be operated with a smaller volume change, meaning that a smaller negative effect occurred on the FO performance. Another key factor affecting the FO-LPM system is mass balance since PSS concentration and LPM membrane performance together influence the entire filtration performance. In this regard, as shown in Fig. 3b, the volume of FO permeate must be always higher than LPM permeate volume. Thus, the FO-LPM process can be consistently operated with the same DS concentration and volume. If the effective membrane areas for both the FO membrane and the LPM are the same, according to the results of Fig. 3a, the volume of DS decreases due to a higher water flux of LPM than FO. However, the effective membrane area of FO was around twice that of the LPM (see details in Methods section). Despite the low water flux in the FO, this can be a beneficial in generating the amount of permeate in a large FO effective membrane area. Therefore, different water fluxes on each side can be solved by regulating the effective membrane area within the FO portion of the process.

## Discussion

PSS, which is a polymer, in high concentrations had high viscosities leading to a more severe ICP. However, this can be reduced by increasing the cross-flow velocity (CFV) or adding a surfactant such as TWIN20. Higher CFVs can lessen the formation of CP layer, and adding surfactants can reduce viscosity and interaction between the polymers or between the membrane surface and polymers^6–8^. During the FO-LPM experiment, magnetic stirrer was used for reducing CP and permeate retention phenomenon by dilution. When dead end filtration experiment or submerged type membrane process was used, agitator or magnetic stirrer could be used for reducing the CP or formation of cake layer^22,23^. Future studies should examine the relationship between viscosity and osmotic pressure, and the effect of CP for the DS in FO process, including the corresponding analysis and its systematic modelling. Moreover, economic evaluation with energy consumption for different viscosities will be conducted in the future study after optimizing the process. Furthermore, it is noted that the severe effects of CP upon the cake layer and convection/diffusion rates of the molecule can occur under high operating pressure. Therefore, a high operating pressure and concentrations of FW can lead to significant deposits and/or the build-up of a fouling layer on the LPM surface. Thus finding the optimal PSS concentration is required for the LPM process. Additionally, an adverse effect of reverse pressure from draw side to feed side was not significant in the FO-LPM process due to the low operating pressure requirement for the LPM process. Therefore, FO and LPM performance within the FO-LPM system was higher when the LPM membrane with low MWCO was used. However, the rejection rate for 10 KDa LPM may be lower, so 3 KDa or 1 KDa LPM membrane should be applied to the FO-LPM process when applying a PSS DS. For 1 KDa and 3 KDa LPMs, where high rejection rates of DS are observed, a lower water flux could be obtained compared to the 10 KDa membrane. However, a higher rejection rate means that the concentration of the DS can be maintained, which is a factor that can increase the possibility of the FO-LPM hybrid system. Based on the data obtained from this study, an additional modelling and optimization will be carried out for its real application. In sum, the FO-LPM hybrid system, which has been designed as a viable energy-saving wastewater treatment and wastewater reuse process, has shown promise with the use of DI water as the FS and PSS solution as the DS. Even though the performance of the FO-LPM process was lower than the singular FO and LPM processes, this experimental approach showed the possibility of its application in industrial wastewater treatment and wastewater reuse. In addition, is suggested that this novel process can present a breakthrough in FO technology that is often limited by high operation and management costs.

## Methods

### Pump-less FO-LPM system set-up and operation

A custom-made lab-scale FO-LPM system was set-up to evaluate the performance of the FO-LPM process. A thin film composite (TFC) FO membrane obtained from Toray Chemical Korea, Inc. was used. It is comprised of a thin and selective polyamide (PA) active layer and a porous polysulfone (PS) support layer. It was selected because this membrane showed the greatest performance in terms of permeate flux among other membranes^19^. The LPM part from the FO-LPM process is operated with the pressure generated from the permeate generated by FO process, which increases the volume of the DS. Therefore the FO membrane having the highest flux was chosen in the study. Three different molecular weight cut-offs (MWCOs -1, 3, and 10 KDa) of cellulose acetate-based UF membranes supplied by Millipore Sigma (Germany) were tested in the LPM. Figure 1 shows a schematic diagram of the FO-LPM process. The effective area for each membrane in the FO and LPM processes were 70 cm^2^ and 32 cm^2^, respectively. The FO portion of the FO-LPM hybrid process was tested using an active layer facing the FS at room temperature (25 ± 1 °C) with the FS’s flow rate kept at 200 mL min^−1^. FO permeate was regenerated by the LPM and 10 KDa, 3 KDa and 1 KDa UF membranes were used in the LPM portion of the process to regenerate PSS. A magnetic stirrer was placed inside the closed DS tank to reduce the phenomenon of concentration polarization (CP) that may occur on the membrane surface. Stirring also served to balance the concentration differences between of the solution produced by the FO portion of the process and that produced by the LPM portion that resulted in high viscosity. DI water, which was used as a FS, was supplied by gear pump (Long Pump WT3000-1FA) into the FO portion of the FO-LPM system. Digital balances (AND GF-6000, USA) located under the FS and permeate tanks were used to check their weight changes. The balance was then connected to a computer that calculated the water flux.

### Preparation of PSS DS

Commercially available PSS (with an average molecular weight of 70,000 g mol^−1^) was purchased from Sigma-Aldrich Co. A PSS solution with a concentration of 0.4 g mL^−1^ was prepared by dissolving 400 g of PSS powder into 1 L of DI water at 25 °C. Although PSS has relatively higher solubility than other polymeric substances, its solubility was not as high as ionic salts and therefore some stirring was required. For stirring, a magnetic stirrer was used. The PSS solutions with concentrations of 0.04, 0.08, 0.10, 0.15, 0.20 and 0.22 g mL^−1^ that were to be tested were prepared by diluting the 0.4 g mL^−1^ PSS solution. The following parameters were measured to analyze the physicochemical characteristics of the PSS solution: polarity, osmotic pressure, conductivity, viscosity and particle size. For the measurements of each parameter, an Osmometer (FISKE 210, Advanced instrument INC, USA), Conductivity meter (Orion 4 Star, Thermo Scientific, USA), Viscometer (Vibro viscometer SV-1A, A&D weighing, USA), and Particle size and solution zeta potential analyzer (Nanotrac Wave II, Microtrac, USA) were used accordingly.

### Analytical methods

The water fluxes in the FO and LPM filtration test were calculated using the following equation:

1 $${J}_{w}=\frac{{\rm{\Delta }}V}{At}$$

where, J_w_ is the water flux of the membrane (L m^−2^h^−1^, LMH), ∆V is the change in permeate volume, A represents the area of the membrane (m^2^), and t is the operating time^7,14,24,25^.
